# HMGA2 immunostaining is a straightforward technique which helps to distinguish pulmonary fat-forming lesions from normal adipose tissue in small biopsies: a retrospective observational study about a series of 13 lung biopsies

**DOI:** 10.1186/s13000-017-0603-x

**Published:** 2017-02-23

**Authors:** Nicolas Piton, Émilie Angot, Florent Marguet, Jean-Christophe Sabourin

**Affiliations:** Department of Pathology, Rouen University Hospital, F 76 000 Rouen, France

**Keywords:** Tracheobronchial lipoma, Bronchial hamartoma, Bronchial biopsy, HMGA2, Immunohistochemistry, Gene translocation

## Abstract

**Background:**

A tracheobronchial lesion observed during an endoscopic examination is usually sampled by the pulmonologist and sent to the pathologist for microscopic examination. Adipocytes may be observed in the lamina propria of tracheobronchial biopsies, which may complicate diagnosis of sampled lesions because these adipose cells may be part of the lesion (lipoma or pulmonary hamartoma), but may also be a normal component of the bronchial mucosa. Because endoscopic samples frequently miss their target, adipocytes observed in such biopsies usually lead to uncertainty regarding diagnosis. Both pulmonary hamartomas and lipomas have a high frequency of translocations involving *HMGA2*, resulting in over expression of the fusion protein. The literature suggests that only 31% of tracheobronchial lipomas are correctly diagnosed on biopsy, sometimes leading to unnecessary aggressive surgical resection.

**Methods:**

We performed retrospective study of tracheo-bronchial biopsies containing adipocytes using HMGA2 immunostaining in order to define their nature and to assess the diagnostic utility of this marker.

**Results:**

In total, 13 lesions biopsied in 12 patients and containing adipocytes were immunostained for HMGA2. Nuclear immunostaining was detected in 7 out of the 13 lesions (54%), allowing us to diagnose a lipoma or hamartoma.

**Conclusion:**

HMGA2 immunostaining is an affordable and straightforward technique for accurate description of biopsies containing adipose cells. When positive, a diagnosis of benign adipose lesion can be made with confidence since well-differentiated liposarcomas have never been described in the tracheobronchial tree. Our work enabled us to diagnose a benign adipose lesion in 54% of cases, above the rate of 31% reported in the literature, based solely on morphological analysis. Overall, HMGA2 immunostaining could help pathologists to provide accurate diagnosis of tracheobronchial adipose lesions, leading to conservative treatment, for the overall benefit of patients.

## Background

**Fig. 1:**
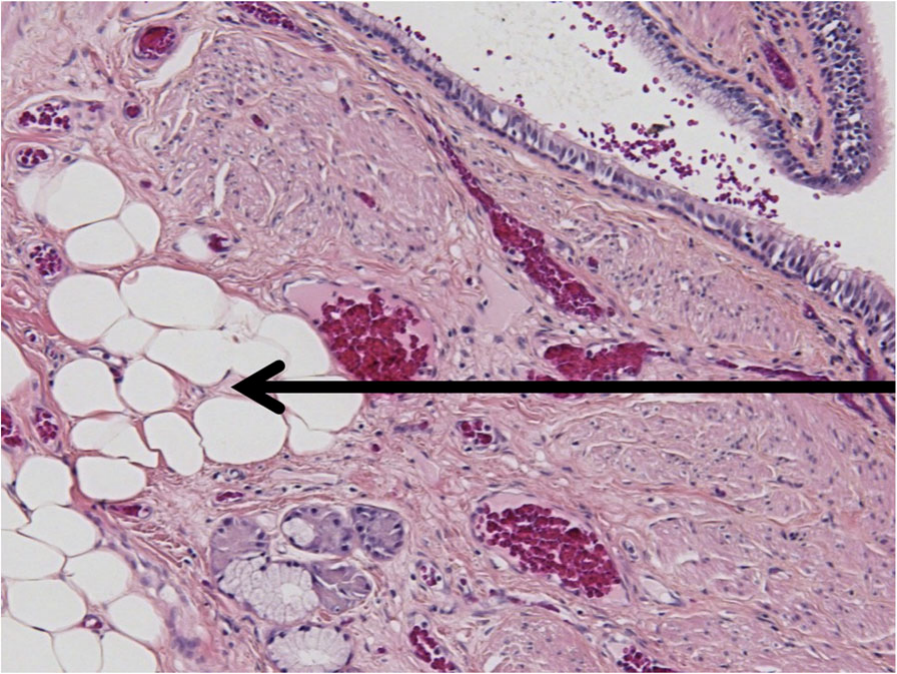
Example of adipose cells in the lamina propria of a normal bronchus. This image was obtained from the bronchial margin of a resection specimen of a lobe for carcinoma. The lamina propria contains adipose cells, just above the cartilaginous ring (*square*). The slide was stained by hematoxylin and eosin. Scale bar 50 μm

**Fig. 2:**
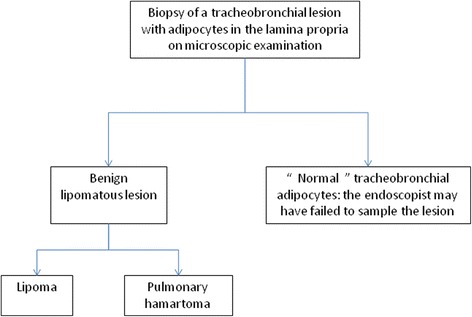
Main hypotheses when adipocytes are observed on tracheobronchial biopsies

A tracheobronchial lesion observed during an endoscopic examination is usually sampled by the pulmonologist and sent to the pathologist for microscopic examination. Adipocytes may be observed in the lamina propria, which may complicate diagnosis of sampled lesions. These adipose cells may be part of the lesion (lipoma or pulmonary hamartoma), but may also be a normal component of the bronchial mucosa (Fig. 1), leading to uncertainty regarding diagnosis because endoscopic samples frequently miss their target (Fig. 2).

Bronchial biopsy demonstrates a low rate of accuracy for the diagnosis of lipoma, 31% according to Muraoka et al. [1]. Therefore, it is crucial to make an accurate diagnosis of lipoma on biopsy, as this allows the pathologist to exclude epithelial malignant lesions and to propose conservative treatment such as endoscopic resection [1, 2].

The tracheobronchial tree is rarely involved in adipose lesions, accounting for 0.1 to 0.5% of all pulmonary lesions [3, 4]. Conversely, pulmonary hamartomas are the most common benign pulmonary neoplasms [5]. Both pulmonary hamartomas [6–9] and lipomas [10, 11] have a high frequency of translocations involving the *HMGA2* (High Mobility Group A2) gene, resulting in over expression of the fusion protein.

This over expression is detectable by immunohistochemical techniques on formalin-fixed paraffin-embedded tissues. The anti-HMGA2 primary antibody (1: 500; 59170AP, Biocheck, Foster City, CA, USA) is a validated clone for detection of HMGA2 expression in formalin-fixed paraffin-embedded tissues [12].

Over the past few years, we have encountered adipose cells on small tracheobronchial biopsies. Given the recent availability of HMGA2 immunostaining, we performed a retrospective analysis to provide an accurate description of these samples. In addition, we assessed the diagnostic utility of HMGA2 immunohistochemistry specifically in cases of tracheobronchial biopsies.

## Methods

### Selection of cases

Cases of tracheobronchial biopsies performed between 01/01/1990 and 01/03/2016 were retrieved from the anatomic pathology database of Rouen University Hospital. Biopsies coded as bronchial hamartoma (24 cases), bronchial lipoma (0 case), tracheal hamartoma (1 case) and tracheal lipoma (1 case) were included in the present study. The corresponding pathology reports were retrieved. Three cases were excluded as the endoscopic appearance was normal (systematic biopsies), but were used as negative controls. To sum up, the criteria for including cases were: 1) a biopsied macroscopic lesion during bronchoscopy and 2) adipose cells in the lamina propria of the biopsy.

Then, the original hematoxylin and eosin stained slides were retrieved and examined by two experienced pathologists (N.P. and J.C.S.). Finally, ten cases were excluded since only cartilaginous or fibrous lesions, but no adipose lesions, were observed.

Demographics and clinical data (age at biopsy, sex and presentation) of patients were extracted from clinical information available in pathology and clinical reports.

Formalin-fixed paraffin-embedded tissue samples of tracheobronchial biopsies were retrieved for immunostaining.

Twenty resection specimens of lung carcinoma with adipose tissue observed on samples of the tumor-free bronchial surgical margins were used as a control for normal adipose tissue from the tracheobronchial tree (example in Fig. 1). This control group was chosen among 124 retrospective resection specimens analyzed in our department in 2015 and 2016.

### Immunohistochemistry

Formalin-fixed paraffin-embedded tissue samples of tracheobronchial biopsies from included cases were retrieved and 4 μm slide sections were cut. Slides were immunostained using the anti-HMGA2 primary antibody (1: 500; 59170AP, Biocheck, Foster City, CA, USA). Deparaffinization, antigen retrieval and immunostaining were performed using a Benchmark ULTRA device (Ventana-Roche; Switzerland). Slides were heated to 72 °C, then incubated during 30 min with the antibody, next rinsed in buffer solution.

Positive control consisted of section of intra-muscular lipoma. Negative control consisted of normal adipose tissue of a resection specimen for skin melanoma.

A lesion was considered as HMGA2 positive when at least one cell nucleus was stained per high-power field, according to Dreux et al. [12].

## Results

In total, 13 lesions from 12 patients were included in our study (Table 1). One patient presented with two distinct lesions sampled during the same bronchoscopy, one in the right lower lobe (lesion 4) and one in the left upper lobe (lesion 5). One lesion (case 9) was sampled twice, one month apart. Mean age at biopsy was 57 years (standard deviation = 19). Seventy-five percent of patients were men (9 cases) and 25% were women (3 cases). Forty-six percent (*n* = 6) of lesions were located in the left lung (2 in the left upper lobe, 4 in the left lower lobe), 38% (*n* = 5) in the right lung (3 in the right upper lobe, 1 in the middle lobe and 1 in the right lower lobe) and only 15% (*n* = 2) were tracheal. Three patients had a history of cancer: case 2 with ENT carcinoma, case 6 with small cell neuroendocrine carcinoma and case 7 with bronchial squamous cell carcinoma. Description on computerized tomography scanner were retrieved in medical records for only 2 lesions (cases 9 and 11).

**Table 1:** Clinical and pathologic characteristics of the 13 included lesions

Case	Sex	Age (years)	Presentation	Location	HMGA2 staining	Remark
1	M	61	Smooth endobronchial lesion.	Right upper lobe	**POSITIVE**	
2	M	65	ENT carcinoma. Hemoptysis. Suspicious endobronchial lesion.	Right upper lobe	**POSITIVE**	
3	M	50	Hemoptysis. Small sessile endobronchial lesion.	Left lower lobe	**POSITIVE**	
4	M	55	COPD and obesity. Acute pulmonary insufficiency. Non suspicious endobronchial lesion.	Right lower lobe	NEGATIVE	Superficial epithelial cells were stained.
5	M	55	COPD and obesity. Acute pulmonary insufficiency. Non suspicious endobronchial lesion.	Left upper lobe	NEGATIVE	Superficial epithelial cells were stained.
6	M	58	Small cell neuroendocrine carcinoma. Endoscopic evaluation after 6 courses of chemotherapy.	Left lower lobe	NEGATIVE	
7	M	64	Squamous cell carcinoma of the left upper lobe. Lipomatous endobronchial lesion.	Middle lobe	**POSITIVE**	Superficial epithelial cells were stained.
8	F	44	Ankylosing spondylarthritis. Dyspnea. Small non suspicious endobronchial lesion.	Trachea	NEGATIVE	Superficial epithelial cells were stained.
9	F	77	Smooth pediculated endobronchial lesion. Fat density on imaging.	Left lower lobe	NEGATIVE	Superficial epithelial cells were stained.
10	M	43	Endobronchial lesion.	Left upper lobe	**POSITIVE**	
11	M	77	Pediculated endobronchial lesion causing complete bronchial obstruction. Calcification on imaging. Adipose lesion macroscopically.	Right upper lobe	**POSITIVE**	
12	F	9	Localized bronchiectasis. Round whitish lesion causing complete bronchial obstruction.	Left lower lobe	NEGATIVE	
13	M	76	Incidental. Endoscopy for exposure to asbestos. Smooth endotracheal lesion with yellowish area macroscopically.	Trachea	**POSITIVE**	

Lesion 4 and lesion 5 were presented by the same patient

*M* male, *F* female, *COPD* chronic obstructive lung disease

**Fig. 3:**
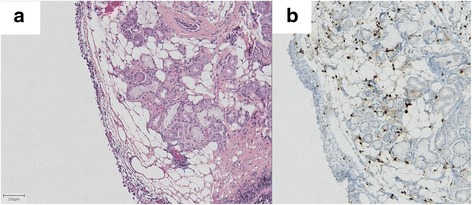
Example of an adipose lesion positive for HMGA2 immunostaining (case 13). This image was obtained from biopsies of a tracheal lesion. Hematoxylin and eosin staining (**a**) and HMGA2 immunostaining counterstained by hematoxylin (**b**). The lesion is located in the lamina propria and consists of sheets of adipose cells surrounding tracheal glands. The nucleus of adipose cells positive for HMGA2 immunostaining. Scale bar 100 μm

**Fig. 4:**
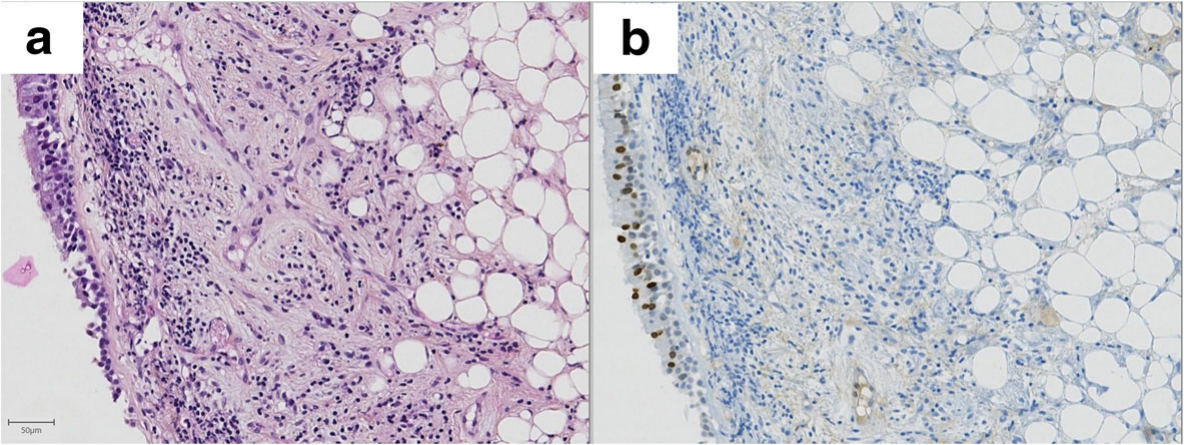
Example of an adipose lesion negative for HMGA2 immunostaining (case 6). This image was obtained from biopsies of a bronchial lesion located in the left lower lobe. Hematoxylin and eosin staining (**a**) and HMGA2 immunostaining counterstained by hematoxylin (**b**). The lesion is located in the lamina propria and consists of sheets of adipose cells. The nucleus of adipose cells negative for HMGA2 immunostaining. In contrast, some nuclei of superficial epithelial cells were positive. Scale bar 50 μm

Nuclear immunostaining was detected in 7 out of the 13 lesions, i.e. in 54% (example in Fig. 3, illustrating case 13). The two lesions presented by the same patient (cases 4 and 5) were both negative and lesion 9, sampled twice, was negative for both specimens. In 6 cases (46%), the nucleus of superficial normal epithelial cells was stained, without any correlation with positivity or negativity of the underlying lesion (example in Fig. 4, illustrating case 6). HMGA2 staining was negative in the adipose tissue observed on the twenty tumor-free bronchial surgical margins of resection specimens of lung carcinoma and on the 3 cases excluded initially because of normal endoscopic appearance.

## Discussion

Normal adipose tissue is not reported in textbooks on normal human histology at the chapter *Lung* [13]. However, experience in our laboratory has shown that adipocytes may sometimes be observed in the bronchial lamina propria (Fig. 1). The presence of adipocytes may complicate accurate description of the biopsied lesion (Fig. 2): is this tissue normal or not? In other words, is it a tracheobronchial lipoma or a pulmonary hamartoma or did the pulmonologist fail to sample the lesion? Indeed, the presence of normal adipose tissue in the lamina propria of the bronchi can sometimes mislead the pathologist into making an erroneous diagnosis of benignity if the endoscopist fails to sample the lesion of interest.

HMGA2 plays a role in mammalian growth [14, 15]. It is reported to be expressed in neoplastic tissues and seems to play a role in cell growth, differentiation and tumorigenesis through modification of chromatine structure [16]. Conversely, according to the literature HMG proteins are not expressed in normal adult tissue [17]. HMGA2 immunostaining is routinely used in soft tissue pathology in order to help to distinguish between normal adipose tissue (HMGA2 negative) and adipose tumor such as lipoma or liposarcomas (HMGA2 usually positive) [12].

According to data previously reported in the literature [1], men were more represented than women in these selected cases (9 out of 12 patients, i.e. 75%) and interestingly, all lesions positive for HMGA2 staining were presented by men only. In accordance with the literature, lesions positive for HMGA2 staining were mainly located in the right lung (4 out of 7 lesions, i.e. 57%), with only 2 cases (29%) in the left lung and 1 in the trachea.

In 6 cases (46%), the nucleus of superficial normal epithelial cells was weakly stained, without any correlation with positivity or negativity of the underlying lesion (example in Fig. 3, illustrating case 6). We believe that this unspecific signal is a potential pitfall and should be kept in mind when handling small, dissociated biopsy cores because it may lead to misdiagnose lipoma or hamartoma if the epithelial nature of the positive cells is overlooked. This faint staining was unexpected as HMGA2 expression is not described in normal adult tissue [17]. Additional research is needed to explain our observation and to determine whether staining was genuine or unspecific.

A radiological description was retrieved in medical records for only two cases [9, 11] and to our knowledge, no patient underwent surgical resection. Therefore, there exists a theoretical risk that included lesions were neither lipoma nor hamartoma. However, for the majority of remaining lesions, the macroscopic appearance favored such a diagnosis, with reassuring terms used by the endoscopisc (Table) and all biopsies contained adipose cells in the lamina propria.

**Fig. 5:**
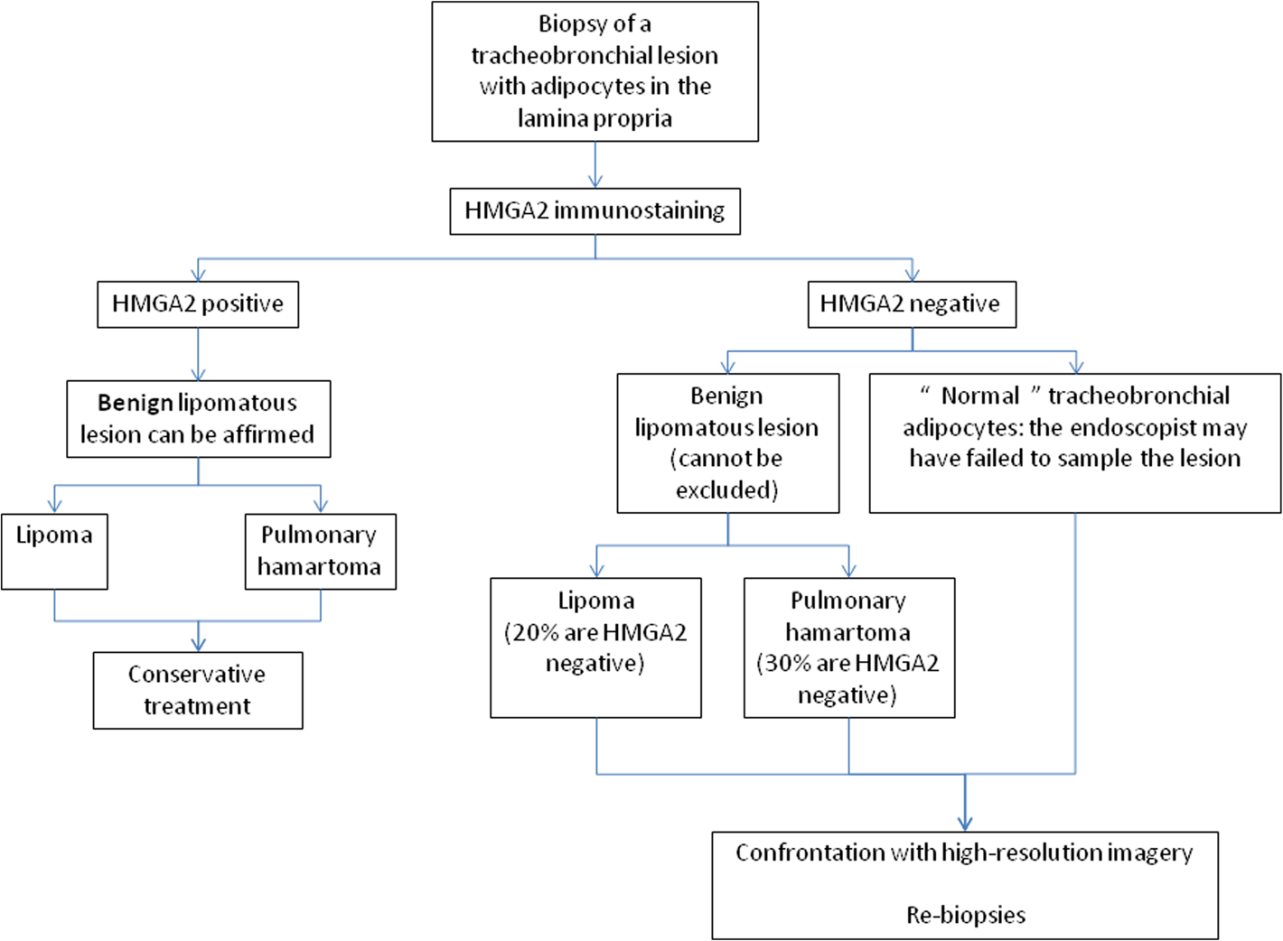
Proposal for an algorithm when adipocytes are observed on tracheobronchial biopsies

To the best of our knowledge, this is the first series of tracheobronchial biopsies containing adipose tissue analyzed by HMGA2 immunostaining. Our work has enabled retrospective confirmation of the lipomatous or hamartomatous nature of the lesions in 54% of cases, above the rate of 31% reported in the literature, based solely on morphological analysis [1]. In this Japanese review, 69% of biopsies led to an erroneous diagnosis, with even 6.25% of cases diagnosed with malignancy, leading to useless and dangerous treatment [1]. The number of cases included in our study is quite small and results should be validated in a larger cohort. However, we are convinced HMGA2 immunostaining is a straightforward and affordable method compared to fluorescent in situ hybridization for accurate description and diagnosis of these rare cases (Fig. 5). When positive, a diagnosis of benign lesion (lipoma or pulmonary hamartoma) can be made with confidence since well-differentiated liposarcomas have never been described in the tracheobronchial tree [18]. Conversely, when negative, uncertainty remains because roughly 20% of lipomas [12] and 30% of pulmonary hamartomas [19] are negative for this marker. This is of great importance since diagnosis of such benign lesions by biopsy significantly impacts treatment.

## Conclusion

In conclusion, we recommend performing HMGA2 staining whenever encountering adipocytes on tracheobronchial biopsies. This could help pathologists to provide accurate diagnosis of benign lesions and lead to conservative treatment, for the overall benefit of patients.

## Abbreviation

HMGA: High Mobility Group A2
